# A rational approach to COVID-19

**DOI:** 10.1186/s40246-020-00300-5

**Published:** 2020-12-22

**Authors:** Ruty Mehrian-Shai

**Affiliations:** Department of Pediatric Hemato-Oncology, Sheba Medical Center, Ramat Gan, Israel

## Abstract

It is crucial to use the wealth of information emerging from the ongoing SARS-CoV-2 pandemic and confront COVID-19 with a rational approach. There are proactive steps to prevent and fight COVID-19. Management of the disease should be according to clinical features and laboratory test markers and personalized therapeutic targets.

## Background

Not applicable

## Main text

In search for a rational approach to COVID-19, one should take into account the following 4 considerations: I. Risk factors, II. Clinical features III, Genomic and Stage markers, and IV. Treatment according to stage.

### Risk factors

People without comorbidities should know that even healthy young people with coronavirus infection may succumb to acute respiratory distress syndrome because of the following risk factors: smoking, stress and depression, low physical activity, and high consumption of saturated fats, sugars, and refined carbohydrates [1]. In addition, underlying medical conditions in any age increase the risk for severe COVID-19 illness. According to the Centers for Disease Control and Prevention, the underlying medical conditions include cancer, chronic kidney disease, chronic obstructive pulmonary disease, heart conditions, immunocompromised state, type 2 diabetes mellitus, obesity (BMI ≥ 30 kg/m^2^), pregnancy, and sickle cell disease.

### Clinical features

Compared to non-severe pneumonia patients, the median age of severe patients is significantly older, and they are more likely to have chronic comorbidities [2]. Most common symptoms in severe patients are associated with, prolonged viral carriage in COVID-19. The symptoms are high fever, anorexia and dyspnea [3], taste and smell loss [4], and diarrhea [5].

### Genomics

Personalized medicine will be enhanced by the integration of advances in human genomics into host big data of genetic determinants of susceptibility and the highly variable clinical manifestations. The host genome influences both innate and adaptive immunity. For example, a genome-wide association study for COVID-19 with respiratory failure detected a susceptibility locus at a chromosome 3p21.31 gene cluster and a potential involvement of the ABO blood-group system in COVID-19 [6]. In this cluster, SLC6A20 encodes the sodium–amino acid (proline) transporter 1 (SIT1) which functionally interacts with angiotensin-converting enzyme 2, the SARS-CoV-2 cell-surface receptor [7]. Interestingly, proinflammatory cytokines interleukin (IL)-6 and tumor necrosis factor (TNF)-alpha stimulate the activity of amino acid transporter system A [8]. C-X-C motif chemokine receptor 6 (CXCR6) regulates the location of lung-resident memory CD8 T cells [9]. CCR1 deficiency increases susceptibility to fatal coronavirus infection [10]. Associations of genes with severe disease were reported for apolipoprotein E, Toll-like receptor 7, and IL-1 signaling pathway [11]. The most significant marker of poor prognosis and high mortality risk in patients with acute respiratory failure due to COVID-19 is vitamin D deficiency [12]. Vitamin D metabolism by vitamin D receptor (VDR) may exert its impact by modulating the innate and adaptive immune responses and suppression of the inflammatory process [13]. Analysis of vitamin D binding protein polymorphisms revealed that vitamin D binding protein1 carriers might be less susceptible to infection and mortality due to COVID-19 [14]. In addition, a positive significant correlation was found between mortality rates and the prevalence of DBP gene polymorphism (GT genotype) at rs7041 locus [15]. Thus, population genomics provides the technology and infrastructure for large-scale health initiatives that are vital in COVID-19 management and in the combat of the virus.

### Stage markers

Patients with moderate symptoms can worsen quickly and hence become much more severely affected, resulting in a high mortality rate [16]. Markers for immune suppression and inflammation should be used to identify the patients that will develop to the severe stage. Lymphopenia is associated with disease severity [17]. White blood cells and neutrophil counts are higher in intensive care unit cases [18]. Neutrophil to lymphocyte ratio may be a good measure to assess the immune suppression [19]. Inflammatory cytokines, tumor necrosis factor and interleukin 6, are higher in the severe stage [20]. Ferritin, which is an inflammation marker, may become the easiest to check for poor prognostic marker and it is high in non-survivors [20]. Once inflammation and immune suppression are detected, proper medication and care may significantly reduce the mortality rate of severe patients.

### Treatment according to stage

Immune-boosting compounds such as interferons [21] and antivirals such as remdesivir [16] are expected to be most beneficial early in disease, when viral replication is still ramping up. On the other hand, in the severe stage which is characterized by immune suppression (loss of T cells) and inflammation (cytokine storm and release inflammatory cytokines), anti-inflammatory intervention would be effective [22]. Particularly, since the nervous system damage caused by viral infection may be mediated by the immune system [22–24], activation of immune cells in the brain will cause chronic inflammation and brain damage, which may be the cause of death. Currently, the recommended treatment is according to the severity of the disease. According to the National Institutes of Health COVID-19 treatment, guidelines panel https://www.covid19treatmentguidelines.nih.gov/, no specific antiviral or immunomodulatory therapy is recommended for patients with COVID-19 who are not hospitalized or who are hospitalized with moderate disease but do not require supplemental oxygen. For hospitalized patients with COVID-19 who require supplemental oxygen, a combination of remdesivir plus dexamethasone or other alternative corticosteroids such as prednisone, methylprednisolone, or hydrocortisone is recommended.

### Proactive steps

In addition to avoiding the exposure to the virus, there are many proactive steps to prevent and/or fight COVID-19. Maintaining a healthy lifestyle and diet is essential along with social distancing, frequent hand washing, etc. The World Health Organization recommends limiting salt (less than 5 g per day), sugar (less than 5% of total energy intake or ~ 5 teaspoons), and total fat intake (less than 30% of total energy intake, of which no more than 10% should come from saturated fat (WHO*). Good hydration with water and avoiding caffeinated, soft drinks, and alcohol as well as 150 min of moderate-intensity or 75 min of vigorous-intensity physical activity per week. Seven to 8 h of sleep is recommended for healthy internal systems. It is recommended to reduce screen time and consume foods containing or promoting the synthesis of melatonin at dinner (almonds, bananas, cherries, and oats) [25]. Healthy and balanced nutrition containing a high amount of minerals, antioxidants, and vitamins can boost immune function [26]. Immune boosting elements can be consumed by sweet potatoes, carrots, and green leafy vegetables (beta carotene) red peppers, oranges, strawberries, broccoli, mangoes, lemons (vitamins C), nuts, seeds, spinach, and broccoli (vitamin E). Vitamin D is important for the healthy respiratory tract, decreasing the production of proinflammatory cytokines. Vitamin D can be gained during sun exposure or food consumption (fish, liver, eggs). Zinc from poultry, red meat, nuts, pumpkin seeds, sesame seeds, beans, and lentils will enhance immunity and inhibit virus proliferation. Food supplements to boost the immune system and reduce inflammation include curcumin, cinnamon, garlic, black pepper, selenium, lactoferrin, and quercetin [27]. Lactoferrin is a protein that is present in the milk exhibits antiviral activity, including against coronaviruses [28]. Selenium is abundantly found in common foods such as corn, garlic, onion, cabbage, and broccoli [27]. Quercetin is a polyphenolic compound, a type of flavonoid which is found in a variety of plants consumed by humans and available as a dietary supplement [29]. These recommendations are general prophylactic and therapeutic support measures. Recently, gut bacterial dysbiosis was linked to poor disease outcome and viral clearance [30]. It is possible to recruit the microbiome for maintenance, homeostasis, and recovery. Short-chain fatty acids (SCFAs) are the products of the fermentation of dietary fibers by the anaerobic intestinal microbiota in the gastrointestinal tract. SCFAs can reduce the luminal pH, enhance the absorption of some nutrients, and reduce plasma glucose and cholesterol levels [31–33]. Consuming dietary fibers and enhancing bacteria population by probiotics consumption including *Bifidobacteria*, *Lactobacillus Faecalibacterium prausnitzii*, *Eubacterium rectale*, *Bacteroidetes*, and *Firmicutes*, *Lachnospiraceae* will regulate the pH of the gut, prevent leaky gut, and combat inflammation.

## Conclusions

Personalized medicine and proactive steps to fight COVID-19 will reduce the severity of symptomatic individuals and the high fatality Toll of SARS-CoV-2.

## Data Availability

Not applicable
